# The undergraduate nursing students’ encountering experience with recovery patients as educators

**DOI:** 10.1192/j.eurpsy.2022.2196

**Published:** 2022-09-01

**Authors:** R.-Y. Gao, E.C.-L. Lin

**Affiliations:** 1 National Cheng Kung University, Nursing, Tainan City, Taiwan; 2 National Cheng Kung University, Taiwan, Department Of Nursing, Tainan City, Taiwan

**Keywords:** Recovery, Qualitative study, Patients as educators, Undergraduate nursing students

## Abstract

**Introduction:**

Nursing students’ attitudes towards mental illness will affect their perception about caring patients with mental illness and their willingness to work in the field of mental health. Evidence supported that contact with recovery patients can change people’s perception of mental illness.

**Objectives:**

The study aims to explore the undergraduate nursing students’ encountering experience with recovery patients as educators.

**Methods:**

A qualitative study using purposive sampling was conducted with undergraduate nursing students in southern Taiwan. Content analysis was used to identify the students’ experience as encountering with the recovery patient as an educator.

**Results:**

As recovery patients participated in class, sharing their recovery journey and learning with students to produce a recovery story, it provided recovery patients and students an equal and mutually beneficial partnership. Four main themes about undergraduate nursing students’ attitudes were identified as. (1)Changing the mindset to patients with mental illness — We are human beings. There’s not much difference between us. (2)Turning positive attitudes towards patients with mental illness — We can compose a better life together! (3)Closing the distance between students and patients with mental illness — I am willing to be close to you. (4)Reflecting and growing in self-understanding and values — I am recovered, too.

**Conclusions:**

This study found that the strategy of recovery patients as educators can improve future nurses’ attitudes towards mental illness, help them deeply learn about patient’ recovery journey. It might beneficial to help students developing their competency in patient-centered care. Future study could examine the effect of the recovery patients as educators.

**Disclosure:**

No significant relationships.

